# From Flying Surgeon to Permanent Hub: A Historical Vignette of Decentralised Neurosurgical Service Development in Rural East Coast Sabah

**DOI:** 10.21315/mjms-02-2026-113

**Published:** 2026-08-31

**Authors:** Teck Cheng Yap, Shze Ee Tan, Win Ner Yap, R. G. Melchior, Jen Nee Bong, Wan Nurul Sakinah Wan Mohd Azaharuddin, Muhammad Naji-Ullah Nur Iman Azlan, Mohd Sofan Zenian

**Affiliations:** 1 Unit of Neurosurgery, Tawau Hospital, Peti Surat 67, 91000 Tawau, Sabah, Malaysia; 2 Department of Surgery, Duchess of Kent Hospital, 90000 Sandakan, Sabah, Malaysia; 3 Department of Surgery, Hospital Lahad Datu, 91100 Lahad Datu, Sabah, Malaysia; 4 Department of Neurosurgery, Hospital Queen Elizabeth, 88200 Kota Kinabalu, Sabah, Malaysia

**Keywords:** global neurosurgery, rural healthcare, decentralisation, teleneurosurgery, flying surgeon, regional neurosurgical model

## Abstract

**Background:**

Access to neurosurgical care in remote regions remains challenging because of the uneven distribution of specialists, transportation limitations, and infrastructure constraints. For many years, patients from East Coast Sabah relied on transfer to Queen Elizabeth Hospital, Kota Kinabalu, for emergency and elective neurosurgical care, often requiring long-distance evacuation. This study describes the phased development of a decentralised neurosurgical service in Tawau and evaluates associated regional service trends.

**Methods:**

This descriptive historical vignette and service evaluation reviewed aggregated neurosurgical data from Tawau Hospital, Duchess of Kent Hospital (Sandakan), and Lahad Datu Hospital from 2019 through April 2026. Service activity was analysed across three phases: the flying surgeon model, rotational specialist postings, and permanent specialist deployment. Referrals, admissions, outpatient attendance, operative activity, interhospital transfers, and mortality were evaluated descriptively.

**Results:**

The East Coast Sabah Neurosurgical Service evolved from a monthly visiting service to rotational specialist coverage and, ultimately, a permanent Tawau-based hub in 2025. Referrals, outpatient attendance, and operative workload increased progressively across the three phases. Mean annual referrals increased from 1,388 to 1,802 and reached an annualised 2,130 during early Phase 3. Annual outpatient attendance increased from 1,117 to 1,675, with an annualised total of 2,526 visits. Operative volume increased from 202 to 366, with an annualised total of 374 procedures, while the proportion of elective procedures rose from 1.9% to 16.5%. Crude inpatient mortality rates were 5.8%, 4.9%, and 3.2% across the three phases. Following establishment of the permanent hub, emergency transfers from Tawau decreased from approximately 35 per year during Phase 2 to an annualised 18 per year during early Phase 3, corresponding to a crude administrative estimate of annual government cost avoidance of approximately RM425,000 to RM680,000 through reduced emergency air evacuations.

**Conclusion:**

The phased decentralisation of neurosurgical services in Tawau was associated with expanded local capacity, improved continuity of care, and reduced reliance on transfers to a tertiary referral centre for selected neurosurgical conditions.

## Introduction

Neurosurgery is among the most resource-intensive specialities in modern medicine, requiring highly specialised personnel, advanced imaging technology, and intensive care facilities. Globally, neurosurgical services are distributed unevenly, with most neurosurgeons concentrated in major urban centres, leaving vast rural regions—where the burden of trauma and stroke is often high—critically underserved (1, 2). As a result, an estimated five billion people lack access to safe, affordable, and timely surgical care, including neurosurgical services (3, 4).

In Malaysia, modern neurosurgery began in the 1960s and gradually expanded across tertiary hospitals nationwide. Neurosurgical services in Sabah were formally established at Queen Elizabeth Hospital (QEH), Kota Kinabalu, in February 2003 under the leadership of Dr. Pulivendhan Sellamuthu, who pioneered modern neurosurgery in the state. Initially staffed by a small team, the department progressively expanded its capabilities in trauma, spinal, and intracranial surgery, becoming Sabah’s primary neurosurgical referral centre.

For nearly two decades, patients from East Coast Sabah—including Tawau, Sandakan, Lahad Datu, Kunak, and Semporna—were routinely transferred to QEH for both emergency and elective neurosurgical care. Tawau and Sandakan Hospitals functioned as intermediary referral centres, although their neurosurgical services remained largely dependent on visiting teams from QEH. Transfers to Kota Kinabalu, often requiring medical evacuation by air, posed substantial logistical challenges, financial costs, and clinical risks. Patients with neurologic instability were particularly vulnerable to deterioration during prolonged transport, resulting in delays to definitive treatment for time-sensitive conditions. In resource-limited settings, long-distance transfer of neurosurgical patients has been associated with increased morbidity, mortality, and health care costs (5, 6).

In response to these challenges, the Ministry of Health Malaysia initiated a phased decentralisation of neurosurgical services to East Coast Sabah. Service development progressed from a visiting “flying surgeon” model to rotational specialist postings and, ultimately, the establishment of a permanent neurosurgical hub at Tawau Hospital in 2025.

This study presents a descriptive historical vignette and service evaluation of neurosurgical decentralisation in East Coast Sabah. Specifically, it describes the phased evolution of neurosurgical services—from a flying surgeon model to rotational specialist postings and ultimately a permanent regional hub—and evaluates associated trends in referrals, admissions, outpatient attendance, operative activity, interhospital transfers, and mortality across successive phases of service development.

## Methods

### Study Design

This study was a descriptive historical vignette and retrospective service evaluation documenting the evolution of neurosurgical services across East Coast Sabah. Aggregated administrative and clinical service data were analysed descriptively to evaluate trends in neurosurgical activity over time. Given the service development nature of the study, no inferential statistical analyses were performed.

### Study Setting and Time Frame

The study was conducted across three hospitals in East Coast Sabah: Tawau Hospital, Duchess of Kent Hospital (Sandakan), and Lahad Datu Hospital. Data collected between 1 January 2019 and 30 April 2026 were included.

The study period encompassed the transition from a visiting neurosurgical service model to the establishment of a permanent neurosurgical hub in Tawau.

### Definition of Service Phases

To facilitate evaluation of service evolution over time, neurosurgical development was categorised into three predefined phases based on the prevailing model of specialist neurosurgical service delivery.

#### Phase 1 (Flying Surgeon Model; January 2019–December 2022)

Monthly visiting neurosurgeons from QEH provided outpatient services and performed selected emergency and elective procedures in Tawau. Because historical records before 2019 were incomplete, analysis of this phase was limited to data available from January 2019 through December 2022.

#### Phase 2 (Rotational Posting Model; January 2023–August 2025)

Neurosurgeons were assigned to Tawau on monthly rotational postings, providing on-site emergency and elective neurosurgical services.

#### Phase 3 (Permanent Hub Model; September 2025–April 2026)

Two full-time neurosurgeons were permanently based in Tawau, providing continuous regional neurosurgical coverage.

### Data Sources

Data were obtained from anonymised hospital administrative systems, neurosurgical referral logs, operating room databases, outpatient clinic registries, mortality audits, and medical evacuation (MEDEVAC) and interhospital transfer records. Only aggregated service data were analysed, and no identifiable patient information was collected.

### Variables Analysed

The study evaluated several key service indicators annually, including neurosurgical referrals, admissions, outpatient attendance (new and follow-up visits), operative workload (emergency and elective procedures), transfers to QEH in Kota Kinabalu, and inpatient neurosurgical mortality. The inpatient mortality rate was calculated using the total number of neurosurgical admissions as the denominator.

Service development milestones, including specialist deployment, equipment acquisition, and imaging availability, were also reviewed. Cost implications were presented as approximate administrative estimates based on reductions in MEDEVAC utilisation and the expansion of local operative capacity.

### Data Analysis

All data were analysed descriptively. Trends across the study period and predefined service phases were examined to evaluate changes in regional neurosurgical activity, operative capacity, referral volume, interhospital transfer patterns, and inpatient mortality.

To facilitate comparisons across service phases of unequal duration, selected indicators were expressed as mean annual values. For periods with incomplete observation windows (e.g., September 2025 through April 2026), annualised estimates were calculated for descriptive comparison only and should be interpreted with caution, particularly for the shorter Phase 3 period. No inferential statistical analyses were performed.

Because data collection began in 2019, findings for Phase 1 reflect only the later portion of the flying surgeon model and may not fully represent earlier service activity. Similarly, Phase 3 data represent the early post-implementation period and should be interpreted cautiously, with emphasis placed on observed service trends rather than definitive conclusions.

### Ethical Considerations

This study was conducted as part of an internal service evaluation and quality improvement initiative. Only anonymised administrative data were reviewed, and no individual patient identifiers were collected. In accordance with institutional and Ministry of Health Malaysia guidelines, formal ethics approval was not required for this retrospective analysis.

## Results

### Evolution of the Neurosurgical Service in Tawau

Before 2023, Tawau received monthly visits from neurosurgeons based at QEH, who conducted outpatient clinics and performed selected procedures, including ventriculoperitoneal shunt insertion, burr-hole drainage of subdural hematoma, and minor tumour resections. This “flying surgeon” model represented the first structured neurosurgical service available to patients in East Coast Sabah. Economically, the model was substantially more cost-effective than repeated patient transfers to Kota Kinabalu, as a single surgeon’s visit could manage multiple patients at a fraction of the cost of repeated medical evacuation flights (7). Clinically, it also reduced patients’ exposure to the risks associated with long-distance transport by road or air, which can be particularly hazardous for individuals with neurologic instability (8). Although short-term visiting surgeon missions have historically been a common strategy for delivering neurosurgical care in underserved regions (9), they are increasingly recognised as a temporary solution with inherent limitations in sustainability and local capacity building. Current global neurosurgery initiatives instead emphasise long-term partnerships, workforce development, and local training to achieve sustainable improvements in access to care. In 2023, the service transitioned to a rotational posting model in which one neurosurgeon from Kota Kinabalu was assigned to Tawau for one month at a time, providing on-site coverage for emergency surgery, inpatient care, and outpatient clinics. This model improved access to neurosurgical care and reduced the need for interhospital transfers. However, it also placed considerable demands on a single rotating neurosurgeon, who frequently served a regional population of more than 1.5 million with limited specialist support, reflecting the workforce challenges commonly encountered in resource-limited settings (10). The rotational nature of the service also reduced continuity of care, as follow-up patients were often reviewed by different neurosurgeons each month, a practice that may adversely affect long-term patient management (11).

Recognising these limitations and the increasing regional demand for neurosurgical services, the Ministry of Health Malaysia approved the establishment of a permanent neurosurgical hub in Tawau. Two full-time neurosurgeons were appointed in September 2025, forming the core of the East Coast Sabah Neurosurgical Service. The hub now provides 24-h neurosurgical coverage for Tawau Hospital and teleconsultation support to Lahad Datu, Kunak, and Semporna. It also shares neurosurgical referral responsibilities for Sandakan with QEH. Under this integrated referral model, complex or high-risk cases from Duchess of Kent Hospital (HDOK) are generally referred to QEH, whereas less complex cases, cerebrospinal fluid shunt procedures, and selected emergency cases may be transferred to Tawau, depending on bed availability and logistical considerations. This arrangement has enhanced referral flexibility and facilitated a more balanced regional distribution of neurosurgical cases. The service also incorporates teleneurosurgery to support clinical decision-making and patient management in remote hospitals (12).

### Demographics and Healthcare Landscape of East Coast Sabah

The East Coast of Sabah, encompassing the districts of Tawau, Sandakan, Lahad Datu, Kunak, and Semporna, is a vast and geographically challenging region (Figure 1). The area is characterised by limited transportation infrastructure, mountainous terrain, and weather conditions that can complicate both ground and air medical transport. The East Coast neurosurgical network serves an estimated regional population of more than 1.5 million people (Table 1).

Table 1 outlines the estimated population, main hospitals, and number of public health clinics within the East Coast region of Sabah. The data illustrate the population density and healthcare infrastructure across five key districts—Tawau, Sandakan, Lahad Datu, Semporna, and Kunak—providing essential contextual background for the development of decentralised neurosurgical services in the region.

Before the establishment of the Tawau Neurosurgical Service, this population relied exclusively on QEH in Kota Kinabalu—located more than 450 km away—for both emergency and elective neurosurgical care. Dependence on a single, distant tertiary referral centre created substantial logistical, clinical, and financial challenges, often resulting in delays in treatment and increased risks associated with long-distance patient transfers. The establishment of a permanent neurosurgical hub in Tawau has expanded regional access to neurosurgical care and reduced reliance on centralised services.

Sabah hosts a substantial population of undocumented migrants, particularly in the coastal districts of Tawau, Sandakan, Lahad Datu, and Semporna (16, 17). These populations often face barriers to timely health care access and contribute to the unique logistical and service delivery challenges encountered in the region (15). The rapid population growth observed in Sabah over recent decades has been linked, in part, to undocumented immigration and has influenced the state’s demographic profile (18).

Health care services in East Coast Sabah are supported by several major hospitals. Tawau Hospital functions as the principal neurosurgical hub for Tawau, Lahad Datu, Kunak, and Semporna, while HDOK in Sandakan serves as a northern referral sub-hub with neurosurgical referral responsibilities shared with QEH. Lahad Datu Hospital functions as a satellite diagnostic centre, while the district hospitals in Kunak and Semporna are integrated into the wider regional referral network.

In addition to hospital services, a network of primary health care clinics provides essential community-based care. Tawau has 24 Klinik Kesihatan (government health clinics) (14), while Sandakan has four (14). Lahad Datu is served by three Klinik Kesihatan Lahad Datu, together with Klinik Kesihatan Tungku and Klinik Kesihatan Felda Sahabat (14). Semporna has two government health clinics and eight government community clinics (15).

### Referrals and Admissions

Progressive increases in neurosurgical referrals and admissions were observed across successive service phases (Table 2). Sandakan consistently accounted for a substantial proportion of the regional referral burden, while Tawau experienced increasing admission volumes following service expansion.

During Phase 1 (2019–2022), a total of 5,551 referrals and 1,468 admissions were recorded across the East Coast network, corresponding to mean annual referral and admission volumes of 1,388 and 367 cases, respectively. During Phase 2 (January 2023–August 2025), mean annual referrals increased to approximately 1,802 cases, while mean annual admissions increased to approximately 540 cases.

Early Phase 3 data (September 2025–April 2026) demonstrated a continued high regional neurosurgical workload, corresponding to annualised estimates of approximately 2,130 referrals and 993 admissions per year. Because these estimates are based on a shorter observation period, they are presented for descriptive comparison only.

### Outpatient Activity

Outpatient neurosurgical activity increased progressively across successive service phases (Table 3). Growth was observed in both new referrals and follow-up visits, reflecting expanded access to specialist neurosurgical care and improved continuity of care across the East Coast network.

During Phase 1, total outpatient attendance was 4,469 visits, corresponding to a mean annual volume of 1,117 visits. During Phase 2, mean annual outpatient attendance increased to approximately 1,675 visits. Early Phase 3 activity corresponded to an annualised estimate of approximately 2,526 outpatient visits per year, driven largely by increased follow-up attendance after the establishment of permanent specialist services.

### Operative Activity

Operative volume increased substantially across successive service phases (Table 4). Emergency procedures remained the predominant component of the neurosurgical workload throughout the study period; however, the proportion of elective procedures increased progressively following service expansion.

During Phase 1, only 15 elective procedures were performed over the four-year period, representing 1.9% of total operative volume. During Phase 2, elective procedures accounted for 7.4% of all operations. Early Phase 3 data demonstrated a further increase in the proportion of elective procedures to 16.5%, suggesting continued maturation of the regional neurosurgical service beyond its initial emphasis on emergency neurotrauma care.

Mean annual operative volume increased from 202 procedures during Phase 1 to 366 procedures during Phase 2 and an annualised estimate of 374 procedures during early Phase 3. The range and complexity of procedures performed locally also expanded, including cranial and spinal tumour surgery, spinal instrumentation, awake craniotomy, aneurysm clipping, arteriovenous malformation (AVM) surgery, and selected skull base procedures.

### Transfers to QEH

Interhospital transfers to QEH remained necessary throughout all service phases, particularly for patients requiring vascular, endovascular, paediatric, skull base, and other highly complex neurosurgical procedures (Table 5).

Mean annual transfer volume increased from 35 cases during Phase 1 to 65 cases during Phase 2, likely reflecting increased regional referral activity, improved case detection, and recognition of more complex conditions requiring tertiary-level management rather than a failure of service decentralisation.

Following the establishment of the permanent neurosurgical hub, transfer patterns changed substantially. During early Phase 3, transfers originating from Tawau decreased markedly, while the case mix shifted towards patients requiring endovascular intervention or specialised paediatric neurosurgical support unavailable within the East Coast network. Consequently, most emergency cranial procedures and selected vascular cases were managed locally.

### Neurosurgical Mortality

Crude inpatient neurosurgical mortality remained stable or improved across successive service phases despite increasing service activity and operative complexity (Table 6). Mortality rates decreased from 5.8% during Phase 1 to 4.9% during Phase 2 and 3.2% during early Phase 3, using neurosurgical admissions as the denominator. These unadjusted descriptive rates should be interpreted with caution because they do not account for differences in case mix, disease severity, comorbidities, or functional outcomes across phases.

At a descriptive level, the observed trend suggests that increasing local operative activity was not associated with an obvious deterioration in short-term inpatient outcomes. However, no inferential statistical analyses were performed, and these findings should not be interpreted as evidence that service decentralisation improved survival.

### Overall Service Evolution and Health Care System Impact

Overall, the East Coast Sabah neurosurgical network demonstrated progressive expansion across successive phases of service development. Mean annual referrals, admissions, outpatient attendance, and operative workload increased steadily as the service evolved from the flying surgeon model to rotational specialist postings and, ultimately, a permanent Tawau-based hub. Although emergency procedures remained the predominant component of the neurosurgical workload, the proportion of elective procedures increased during the later phases, suggesting a gradual expansion from emergency stabilisation towards more definitive neurosurgical care. Over the same period, crude inpatient neurosurgical mortality remained stable or improved, decreasing from 5.8% during the flying surgeon phase to 4.9% during the rotational posting phase and 3.2% during the early permanent hub phase. These unadjusted rates should be interpreted with caution because they do not account for differences in case mix, disease severity, comorbidities, or functional outcomes. At a descriptive level, the observed trend suggests that increasing local operative activity was not associated with a marked deterioration in short-term inpatient outcomes. However, no inferential statistical analyses were performed, and a formal evaluation incorporating risk adjustment, severity scores, and functional outcome measures would be required to determine the true impact of service decentralisation on patient outcomes.

Following establishment of the permanent Tawau-based neurosurgical hub, transfers originating from Tawau to QEH decreased markedly. During this period, most emergency craniotomies, large intracranial tumours, and selected aneurysm clipping procedures were performed locally. Remaining transfers primarily involved patients requiring endovascular intervention or specialised multidisciplinary paediatric care unavailable within the East Coast network.

The range and complexity of procedures performed locally also expanded, including awake craniotomy, aneurysm clipping, AVM surgery, skull base surgery, and complex spinal instrumentation, reflecting the progressive maturation of regional neurosurgical capability.

The decentralisation of neurosurgical services to Tawau also had important health care system and administrative implications. Before the establishment of permanent neurosurgical coverage and microsurgical capability, patients requiring advanced cranial and vascular procedures were frequently transferred to QEH, Kota Kinabalu, located more than 450 km away. These transfers often required MEDEVAC or, less commonly, ground ambulance transport, resulting in substantial direct costs and logistical challenges.

Between 2019 and September 2025, 149 emergency evacuations were recorded across the East Coast network. The vast majority were conducted by air because the approximately 450-km distance between Tawau and Kota Kinabalu, combined with mountainous terrain and limited road infrastructure, makes ground ambulance transport impractical for most emergency neurosurgical cases. Air evacuation is also vulnerable to weather-related disruptions, which may delay definitive neurosurgical treatment or necessitate prolonged and potentially hazardous land transport.

Following establishment of the permanent neurosurgical hub, emergency transfers originating from Tawau decreased from approximately 35 cases per year during Phase 2 to an annualised 18 cases per year during early Phase 3, representing a reduction of approximately 17 emergency transfers annually. Based on estimated MEDEVAC costs of approximately RM25,000 to RM40,000 per air evacuation, with ground ambulance transfers generally incurring lower costs, this reduction corresponds to a crude administrative estimate of annual government cost avoidance of approximately RM425,000 to RM680,000. These estimates are descriptive and should not be interpreted as a formal economic evaluation because they do not include indirect costs, productivity losses, or long-term infrastructure and equipment maintenance expenditures.

In addition to the direct financial implications, the reduction in emergency air evacuations has important clinical significance. Managing an increasing proportion of emergency neurosurgical cases locally reduces patients’ exposure to the risks associated with long-distance aeromedical transport, including physiologic deterioration during transfer, turbulence-related complications, and delays in definitive treatment caused by adverse weather or logistical constraints. This likely improves patient safety and timeliness of care.

Additional indirect economic benefits were also observed as more patients received emergency and elective neurosurgical care within Tawau. Previously, patients requiring elective tumour, spinal, or cranial surgery travelled independently to Kota Kinabalu, incurring substantial out-of-pocket expenses. The increasing availability of elective procedures locally, made possible by the presence of full-time neurosurgeons, has likely reduced this financial burden while improving timely access to care and continuity of treatment. The ability to perform complex procedures locally, including microsurgical tumour resection, spinal instrumentation, and aneurysm clipping, also optimises the use of operating rooms, anaesthesia services, and inpatient facilities within the East Coast network, reducing reliance on tertiary referral centres. Collectively, these changes reduce transfer-related costs and logistical burdens for both the health care system and patients.

Although often overlooked, these indirect and opportunity costs contribute substantially to the overall health care burden in regions lacking specialised neurosurgical services (1). The ability to manage selected complex cases locally has improved accessibility, enhanced patient convenience, and supported more efficient use of regional health care resources. These findings are consistent with global evidence that regionalised care models in underserved settings can achieve substantial cost savings and improve access to care despite considerable initial investment (19, 20).

## Discussion

### Predicted and Emerging Benefits

The development of a regional neurosurgical hub in Tawau represents an important step towards improving equity, access, and efficiency within Sabah’s health care system. The most immediate and measurable benefit has been improved access to timely neurosurgical care. Before the establishment of the permanent service, patients with emergency neurosurgical conditions, such as intracerebral haemorrhage, traumatic brain injury, or acute hydrocephalus, were either transferred to Kota Kinabalu or, in urgent circumstances, managed locally by general surgeons when they considered it clinically appropriate. Although these surgeons demonstrated commendable initiative in life-saving situations, outcomes were inherently variable because of limited neurosurgical training and the absence of specialised equipment (21). With the establishment of permanent neurosurgical services in Tawau, high-risk emergency procedures—including burr-hole drainage, decompressive craniectomy, ventriculostomy, shunt revision, and emergency craniotomy—are increasingly being performed under direct neurosurgical supervision. This development has likely improved case selection, procedural oversight, and access to definitive specialist care. By reducing delays associated with interhospital transfer, local neurosurgical services may facilitate earlier intervention for time-sensitive conditions (22, 23). Previous studies have likewise demonstrated that timely diagnosis and shorter time to surgery are important advantages of locally available neurosurgical services (24).

The decentralisation of neurosurgical services has also generated important economic benefits. Following establishment of the permanent neurosurgical hub, transfers originating from Tawau declined substantially as an increasing proportion of complex cases—including emergency cranial procedures, large intracranial tumours, and selected aneurysm clipping procedures—were managed locally. This change has reduced transportation costs while improving the efficiency of resource utilisation across both referring and receiving hospitals. Patients and their families have also been spared many of the financial and psychosocial burdens associated with long-distance travel, accommodation, and prolonged absence from work (25). Importantly, the evolution of the service has not simply reduced the number of transfers but has transformed their purpose. A broader range of complex neurosurgical cases is now managed locally, whereas transfers are increasingly reserved for highly specialised services, such as endovascular procedures and selected paediatric neurosurgical conditions that remain unavailable within the East Coast network. This shift has the potential to improve patient outcomes by avoiding unnecessary interhospital transfers for an expanding range of neurosurgical conditions.

Another important benefit relates to patient safety. Long-distance medical transport, whether by road or air, carries intrinsic risks. Sabah’s mountainous terrain and unpredictable weather frequently complicate patient transfers, exposing both patients and health care personnel to the risks associated with potential accidents, turbulence, and transport-related equipment failure (26). Patients with critical neurologic conditions are particularly susceptible to deterioration during interhospital transfer, including haemodynamic instability, neurologic decline, and even in-transit complications (26, 27). Managing a greater proportion of cases locally may reduce these risks while allowing critical care resources to remain focused on patient care within the referring institution rather than prolonged transport missions.

The permanent presence of neurosurgeons has also strengthened regional clinical capacity. Local medical officers, anesthesiologists, nurses, and allied health professionals have gained greater exposure to neurosurgical procedures, perioperative care, and postoperative management, fostering multidisciplinary collaboration (24). This transfer of knowledge and skills may gradually reduce dependence on visiting specialists while supporting the long-term development of regional neurosurgical expertise (28). Capacity-building initiatives, including structured education and training programmes for local health care professionals, are recognised as essential components of sustainable neurosurgical development (11). Over time, the availability of specialist neurosurgical expertise in Tawau may also encourage earlier referrals from district hospitals, as referring clinicians gain confidence that patients can receive timely, definitive care closer to home.

The availability of computed tomography (CT) and magnetic resonance imaging (MRI) in both Tawau and Sandakan further strengthens regional neurosurgical services. Advanced neuroimaging enables more accurate diagnosis of aneurysms, tumours, and spinal disorders that were previously difficult to identify in peripheral hospitals (29, 30). Earlier diagnosis facilitates timely surgical intervention while supporting preoperative planning for increasingly complex elective procedures, including aneurysm clipping and tumour resection, performed within Tawau. The expanding availability of advanced imaging, together with growing surgical expertise, has enabled the East Coast service to manage an increasingly diverse range of cranial and spinal neurosurgical conditions locally, an important factor in improving neurosurgical care in underserved regions (31).

Finally, establishment of the Tawau Neurosurgical Hub has important social and psychological implications. Patients can increasingly receive specialist care closer to home, reducing the need for prolonged separation from their families during treatment. Receiving care within their own communities may strengthen public confidence in the health care system while supporting the Ministry of Health Malaysia’s broader goal of equitable access to high-quality health care.

### Anticipated Challenges

Despite the substantial progress achieved, several important challenges remain in ensuring the long-term sustainability and scalability of the regional neurosurgical service. The most immediate challenge is workforce capacity. Two neurosurgeons currently provide specialist coverage for Tawau, Lahad Datu, Kunak, and Semporna, while sharing neurosurgical referral responsibilities for Sandakan with QEH. Their responsibilities encompass emergency surgery, elective procedures, outpatient clinics, teleconsultation, administrative duties, and regional service coordination, creating a workload that may be difficult to sustain over the long term (32). Without additional staffing or a structured rotational support system involving QEH and HDOK, the risks of fatigue and professional burnout are likely to increase, reflecting global concerns regarding neurosurgical workforce shortages, particularly in rural and underserved regions (32).

A second major challenge is infrastructure and technological capacity. Although Tawau and Sandakan now have CT and MRI facilities, important gaps remain in specialised neurosurgical equipment, including a Cavitron Ultrasonic Surgical Aspirator, image-guided surgical navigation systems, and neuroendoscopy equipment. Complex tumour resections and cerebrovascular procedures require not only technical expertise but also advanced surgical technology, high-quality visualisation, and reliable hemostatic instruments (1). In the absence of these resources, selected complex cases must continue to be referred to Kota Kinabalu, limiting the range of procedures that can be safely performed locally. Similar limitations have been recognised as major barriers to comprehensive neurosurgical care in resource-limited settings (1).

Critical care infrastructure represents another important challenge. The absence of a dedicated neuro-intensive care unit (neuro-ICU), together with limited ventilator capacity at Tawau Hospital, restricts the management of patients with severe traumatic brain injury, complex postoperative care needs, or other critical neurologic conditions. Similar deficiencies in neurocritical care infrastructure have been associated with increased postoperative morbidity and mortality in other resource-limited settings, even when surgical procedures are technically successful (25). Establishment of a dedicated neuro-ICU with appropriate staffing and equipment would likely strengthen postoperative care and facilitate the safe management of more complex neurosurgical cases.

Logistical and supply chain constraints also remain significant barriers. Procurement of essential neurosurgical consumables—including aneurysm clips, bipolar forceps, hemostatic agents, and specialised sutures—may be delayed by centralised purchasing processes and a limited number of local suppliers. Equipment maintenance presents an additional challenge, as biomedical engineering support in regional hospitals is often limited, resulting in prolonged downtime when critical equipment requires repair. These supply chain vulnerabilities are widely recognised barriers to the delivery of effective surgical care in resource-constrained settings (10).

Finally, recruitment and retention of specialist personnel will be critical to sustaining long-term progress. Extended service in remote locations without adequate academic engagement, career advancement opportunities, or research support may discourage long-term retention of neurosurgeons (33). Developing a structured workforce strategy that includes incentives for rural practice, continuing professional development, protected academic opportunities, and collaboration with universities and tertiary referral centres will be essential to attracting and retaining skilled neurosurgical personnel in Tawau and other underserved regions (33).

### Integration with Innovative Models

Several complementary service models may further support the long-term sustainability of neurosurgical care in East Coast Sabah. Teleneurosurgery, first implemented in

Malaysia in 2006, provides a practical strategy for overcoming geographic barriers and specialist workforce shortages. By enabling secure transmission of CT and MRI images through a Digital Imaging and Communications in Medicine (DICOM)-based network, teleneurosurgery allows neurosurgeons in Tawau to review cases from Lahad Datu, Kunak, and Semporna in real time. Experience from Hospital Sultanah Aminah, Johor Bahru, demonstrated that this approach reduced unnecessary interhospital transfers by 37% and eliminated nearly 10,000 kilometres of ambulance travel within a four-month period (12).

Despite the establishment of a permanent neurosurgical hub, the geographic expanse of East Coast Sabah continues to necessitate periodic outreach to smaller hospitals for outpatient clinics, postoperative follow-up, and surgical education. A mobile outreach, or “flying neurosurgeon,” model may therefore remain a valuable extension of the permanent hub. Regular outreach visits can support specialist consultations, postoperative care, and local skills transfer, particularly in geographically dispersed regions (31, 34). Unlike the earlier monthly visiting service, these outreach activities would function as complementary extensions of the permanent hub rather than temporary substitutes, thereby strengthening continuity of care and reinforcing the regional neurosurgical network (31).

A hybrid regional network integrating permanent neurosurgeons, teleconsultation, and periodic outreach may represent the most practical model for Sabah’s geographic context. Under this framework, Tawau would function as the central neurosurgical hub, coordinating with HDOK in Sandakan as the northern referral sub-hub and Lahad Datu Hospital as a satellite diagnostic centre. Such a network would distribute patient workload, facilitate resource sharing, and maintain continuity of neurosurgical services across East Coast Sabah. Similar hub-and-spoke models are increasingly recognised as effective approaches for delivering specialised care in geographically challenging and resource-limited settings (35).

Beyond clinical service delivery, integrating telemedicine into education and workforce development could further enhance long-term sustainability. Teleconferencing platforms may facilitate multidisciplinary team meetings, educational conferences, and virtual case discussions, enabling clinicians in regional hospitals to remain connected with tertiary referral centres and national neurosurgical societies (28). Over time, these activities may strengthen regional capacity and foster closer collaboration with tertiary referral centres and national neurosurgical organisations (28, 33).

### Future Prospects

The establishment of the Tawau Neurosurgical Hub has laid the foundation for continued growth and service expansion. A key future priority is the development of a dedicated neuro-ICU equipped with advanced monitoring capabilities and ventilatory support. Specialised neuro-ICUs have consistently been associated with lower mortality, improved functional outcomes, and reduced health care costs for patients with severe neurologic conditions, including intracerebral haemorrhage, aneurysmal subarachnoid haemorrhage, and traumatic brain injury (36, 37). In addition, hospitals without neurosurgical services, and often without dedicated neuro-ICUs, have reported significantly higher ICU and in-hospital mortality among patients with traumatic brain injury (38). Establishing a dedicated neuro-ICU in Tawau could facilitate safer management of complex postoperative patients, severe neurotrauma, and other critical neurosurgical emergencies while reducing dependence on general ICU capacity.

Expansion of intraoperative technologies, including a surgical microscope, a Cavitron Ultrasonic Surgical Aspirator, neuronavigation systems, and fluorescence-guided imaging, may further enhance the scope and safety of neurosurgical practice (25, 39). These technologies could support the local management of increasingly complex cranial, cerebrovascular, and skull base procedures while reducing dependence on referrals to tertiary centres.

Alongside technological advancement, establishment of a Sabah Neurosurgical Registry would provide a comprehensive platform for monitoring clinical outcomes, complications, procedural trends, and service performance across neurosurgical units throughout the state. Such registries are essential for supporting evidence-based practice, quality improvement initiatives, and benchmarking against national and international standards (40–42).

Academic collaboration represents another important opportunity for future development. Partnerships with local and international universities could formalise clinical teaching rotations, facilitate collaborative research, and support postgraduate neurosurgical training. Such initiatives would strengthen long-term workforce development and regional neurosurgical capacity building (24, 43).

Emerging technologies, including artificial intelligence-assisted imaging triage and advanced telemedicine platforms, may further enhance neurosurgical care delivery in remote settings. However, these technologies should currently be regarded as complementary tools and will require further validation, infrastructure development, and careful implementation before widespread adoption in Sabah (44–48).

With sustained policy support, strategic investment in infrastructure, and long-term workforce planning, the East Coast Sabah Neurosurgical Service has the potential to serve as a regional model for decentralised tertiary neurosurgical care in geographically challenging and resource-limited settings (49, 50).

### Limitations

This study has several limitations. First, it was a retrospective analysis based on aggregated administrative and service-level data, which may contain documentation inconsistencies and did not permit adjustment for patient-level clinical variables, including disease severity, comorbidities, functional outcomes, or long-term neurologic status. Consequently, the observed trends in mortality and operative activity should be interpreted as indicators of service evolution rather than direct prognostic comparisons of patient outcomes. In particular, the reported mortality rates are crude and unadjusted. Without adjustment for case mix, disease severity, or functional outcomes, these rates should not be interpreted as measures of service quality or effectiveness. Future studies incorporating risk adjustment and long-term functional outcome measures will be required to determine the true impact of service decentralisation on patient outcomes.

Second, important process and patient-centred outcome measures—including time from presentation or referral to surgery, transfer time to QEH, length of hospital stay, postoperative complications, discharge functional status, and long-term follow-up—were not consistently available across participating hospitals and service phases. As a result, the clinical impact of service decentralisation beyond overall service activity and mortality trends could not be fully evaluated.

Third, the phased analysis reflects the gradual evolution of the service rather than the implementation of a single discrete intervention. In particular, Phase 3 represents an early post-implementation period with partial-year data, and annualised estimates were presented for descriptive comparison only.

In addition, changes in service delivery during the earlier phases, including the transition from the flying surgeon model to rotational specialist postings, may have influenced referral patterns, operative activity, continuity of care, and data completeness.

Fourth, the economic evaluation was limited to transfer patterns and estimated MEDEVAC expenditures. It did not include a comprehensive health economic analysis incorporating indirect societal costs, productivity losses, or long-term infrastructure, equipment maintenance, and workforce expenditures. Accordingly, the financial estimates presented should be regarded as approximate administrative cost implications rather than a formal economic evaluation.

Finally, differences in infrastructure, imaging availability, staffing, and operative capability among Tawau, Sandakan, and Lahad Datu Hospitals may have influenced referral patterns and case selection, thereby limiting direct comparisons across centres.

Despite these limitations, this study provides valuable insight into the feasibility, operational evolution, and regional health care system impact of neurosurgical decentralisation in a geographically challenging setting.

## Conclusion

The establishment of the East Coast Sabah Neurosurgical Service Hub in Tawau represents an important milestone in the evolution of neurosurgical care in Sabah. From its origins as a monthly flying surgeon service, through an intermediate phase of rotational specialist postings, to the establishment of a permanent neurosurgical team in 2025, the programme demonstrates how a phased approach to decentralisation can help address geographic and logistical barriers to specialised neurosurgical care in underserved regions (24, 35).

With the availability of CT and MRI in Tawau and Sandakan, together with CT capability in Lahad Datu, the East Coast network is increasingly equipped to manage not only emergency neurotrauma but also a broader range of elective cranial and spinal neurosurgical conditions locally. The expansion of local operative capability has been associated with reduced reliance on long-distance interhospital transfers for selected patients while improving access to specialist care and continuity of care for patients and their families (3, 20).

Sustained progress will require continued investment in workforce development, neurocritical care infrastructure, advanced operative technologies, and regional support systems, including teleneurosurgery and structured outreach services (12). Although important challenges remain, the Tawau model provides valuable insight into the feasibility and operational impact of regional neurosurgical decentralisation in geographically challenging settings and may serve as a useful reference for similar health care systems throughout Malaysia and the wider Southeast Asian region.

## Figures and Tables

**Figure 1:**
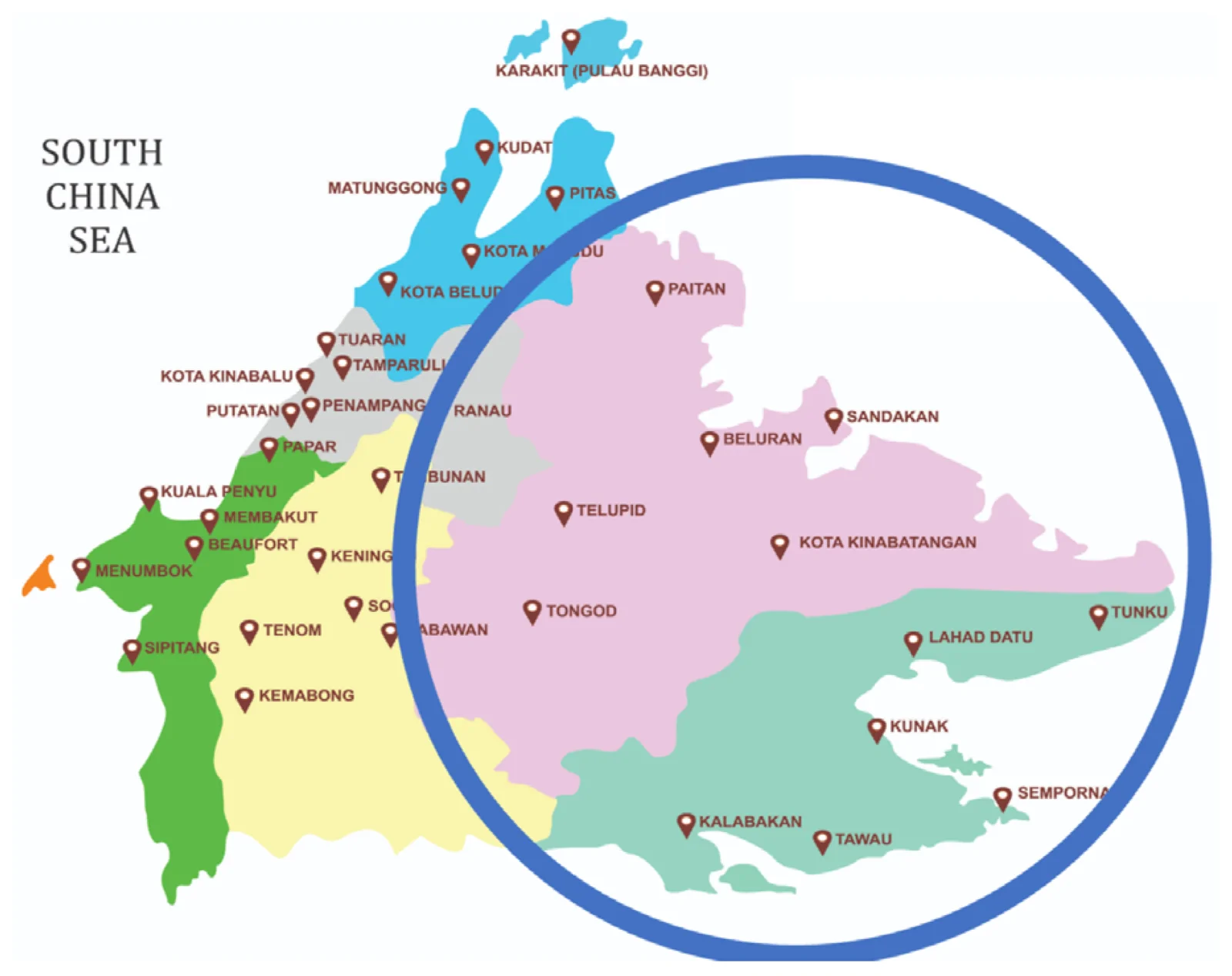
Map of Sabah highlighting the East Coast region covered by the Tawau Neurosurgical Service. The circled area illustrates the broader East Coast Sabah neurosurgical network. Tawau Hospital functions as the principal regional hub for Tawau, Lahad Datu, Kunak and Semporna, while neurosurgical referral responsibilities for Sandakan are shared with Queen Elizabeth Hospital.

**Table 1:** Demographic and healthcare facility distribution across the East Coast districts of Sabah.

Region	Estimated population^a^	Main hospitals	Number of public health clinics
Tawau	697,150	Hospital Tawau	24^b^
Sandakan	535,365	Hospital Duchess of Kent	4^b^
Lahad Datu	212,550	Hospital Lahad Datu	5^b^
Semporna	88,259	Hospital Semporna	2^c^
Kunak	17,035	Hospital Kunak	Not specified in sources

a Fong et al. (13);

b Kaur et al. (14);

c Harris et al. (15)

**Table 2:** Phase-based neurosurgical referrals and admissions across the East Coast network.

Phase	Duration	Tawau	Sandakan	Lahad Datu	Total	Mean annual
**Referrals**						

Phase 1^a^	4 years	2,342	3,044	165	5,551	1,388/year
Phase 2^b^	32 months	2,355	2,300	151	4,806	1,802/year
Phase 3^c^	8 months	724	527	169	1,420	2,130/year*

**Admissions**						

Phase 1^a^	4 years	566	821	81	1,468	367/year
Phase 2^b^	32 months	643	724	74	1,441	540/year
Phase 3^c^	8 months	325	305	32	662	993/year*

a 2019–2022;

b 2023–Aug 2025;

c Sept 2025–Apr 2026;

* Annualised equivalent based on early Phase 3 activity and presented for descriptive comparison only.

**Table 3:** Phase-based outpatient neurosurgical attendance.

Phase	Duration	Tawau	Sandakan	Lahad Datu	Total	Overall attendance	Mean annual attendance
**New cases**							

Phase 1^a^	4 years	104	148	44	296	4,469	1,117/year
Phase 2^b^	32 months	198	171	115	484	4,466	1,675/year
Phase 3^c^	8 months	64	60	23	147	1,684	2,526/year*

**Follow-up cases**							

Phase 1^a^	4 years	1,720	2,112	341	4,173		
Phase 2^b^	32 months	1917	1,528	537	3,982		
Phase 3^c^	8 months	892	524	121	1,537		

a 2019–2022;

b 2023–Aug 2025;

c Sept 2025–Apr 2026;

* Annualised equivalent based on early Phase 3 activity and presented for descriptive comparison only.

**Table 4:** Phase-based neurosurgical operative activity.

Phase	Duration	Tawau	Sandakan	Lahad Datu	Total	Total operations	Mean annual operations	Elective proportion
**Elective**								

Phase 1^a^	4 years	13	0	2	15	809	202/year	1.9%
Phase 2^b^	32 months	38	24	10	72	975	366/year	7.4%
Phase 3^c^	8 months	32	9	0	41	249	374/year*	16.5%

**Emergency**								

Phase 1^a^	4 years	357	398	39	794			
Phase 2^b^	32 months	454	394	55	903			
Phase 3^c^	8 months	142	62	4	208			

a 2019–2022;

b 2023–Aug 2025;

c Sept 2025–Apr 2026;

* Annualised equivalent based on early Phase 3 activity and presented for descriptive comparison only.

**Table 5:** Phase-based transfers to QEH.

Phase	Duration	Tawau	Sandakan	Lahad Datu	Total	Total transfers	Mean annual transfers
**Emergency transfers**							

Phase 1^a^	4 years	30	26	9	65	140	35/year
Phase 2^b^	32 months	39	47	6	92	172	65/year
Phase 3^c^	8 months	5	5	2	12	21	32/year*

**Elective transfers**							

Phase 1^a^	4 years	35	30	10	75		
Phase 2^b^	32 months	28	47	5	80		
Phase 3^c^	8 months	4	4	1	9		

a 2019–2022;

b 2023–Aug 2025;

c Sept 2025–Apr 2026;

* Annualised equivalent based on early Phase 3 activity and presented for descriptive comparison only;

QEH = Queen Elizabeth Hospital.

**Table 6:** Phase-based neurosurgical mortality.

Phase	Duration	Mortality				

		Tawau	Sandakan	Lahad Datu	Total	Rate (denominator: admissions)
Phase 1^a^	4 years	34	42	9	85	5.8%
Phase 2^b^	32 months	31	39	1	71	4.9%
Phase 3^c^	8 months	14	6	1	21	3.2%

a 2019–2022;

b 2023–Aug 2025;

c Sept 2025–Apr 2026.

## Data Availability

The datasets generated and/or analysed during the current study were derived from institutional administrative systems and are not publicly available because of Ministry of Health Malaysia data governance policies. De-identified data may be made available from the corresponding author upon reasonable request and subject to appropriate institutional approval.
